# Biomineralisation and metal sequestration in a crustacean ectoparasite infecting the gills of a freshwater fish

**DOI:** 10.1007/s00360-023-01489-2

**Published:** 2023-05-11

**Authors:** Lutfiyya Latief, Beric M. Gilbert, Annemariè Avenant-Oldewage

**Affiliations:** Department of Zoology, University of Johannesburg, P. O. Box 524, Auckland Park, 2006 South Africa

**Keywords:** Acid mine drainage, Bioindicator, *Clarias gariepinus*, *Lamproglena clariae*, Life below water

## Abstract

It has been suggested that parasites are effective bioindicators as they are sensitive to environmental changes and, in some cases, accumulate trace elements in higher concentrations than their hosts. Accumulated elements sequester in different organs. In monogenean and crustacean ectoparasites, sclerotised structures and egg yolk appear to be the preferred site for element sequestration. In this study, the sequestration of trace elements; Mg, Al, Ca, Fe, Cu, and Zn in *Lamproglena clariae* was studied from two rivers. Adult *L. clariae* were collected from the gills of *Clarias gariepinus* from Lake Heritage in the Crocodile River and in the Vaal River below the Vaal Dam, South Africa. Collected parasites were flash frozen in liquid nitrogen and sectioned with a cryomicrotome. Sections were treated with Phen-Green to observe fluorescent signals. Trace elements in the parasite were analysed using a scanning electron microscope with an energy-dispersive spectroscope (SEM–EDS). Results showed more intense fluorescence signals in the exoskeleton compared to tissues, and in the egg yolk. Analysis by SEM–EDS confirmed the presence of elements in the parasite from both sites. Levels of Al were higher in *L. clariae* from the Vaal River than those from Lake Heritage, and Fe was higher in *L. clariae* from Lake Heritage. Element distribution patterns in the parasite matched those in the water from the sites. Unlike other crustaceans, regulation of metals in adult females of *L. clariae* does not occur through moulting, but high levels occurred in the yolk.

## Introduction

Aquatic organisms are exposed to a mixture of trace elements and chemicals which are of natural and anthropogenic origin. Some elements may become toxic when accumulated above the threshold (Hassaan et al. 2016). As a counter mechanism, organisms regulate levels by binding (or sequestering) elements to proteins in organs or inert tissues, such as bone in vertebrates or the exoskeleton of invertebrates (Mertz 1981; Seymore et al. 1995; Degger et al. 2009; Haug et al. 2011; Martiniaková et al. 2012; Callender 2013). Metal sequestration studies have shown that sequestration patterns are variable between species of organisms (Weeks et al. 1992) and between metal species (Kataria et al. 2022). One of the principal elements found in the hard structures of organisms is calcium. Specifically, in invertebrates the exoskeleton is variably mineralised with calcium carbonate (Bentov et al. 2016). Some metals are able to substitute calcium in the exoskeleton, and in this way, the structure is a main sequestration site for metals (Mergelsberg et al. 2019).

In aquatic and terrestrial invertebrates, sclerotised structures of the exoskeleton such as ovipositors (Quicke et al. 1998), mandibles (Schofield et al. 2002), and tarsal claws (Schofield et al. 2003) sequester higher levels of trace elements including metals. In crustaceans, a significant fraction of the element burden is present in the exoskeleton; distributed between the exoskeleton and the inner matrix (Zanders and Rojas 1996; Munger and Hare 1997). Therefore, trace elements in the exoskeleton are lost during moulting, and this has been suggested as an important regulatory pathway in these organisms (Weeks et al. 1992; Keteles and Fleeger 2001; Bergey and Weis 2007).

Studies on metal accumulation in parasites have shown that some parasites accumulate metals in higher concentrations than in their hosts’ tissues, and because of this, they are considered to be effective bioindicators (Sures 2004; Retief et al. 2006; Nachev et al. 2013; Sures et al. 2017; Pretorius and Avenant-Oldewage 2021). In some instances, parasites are used as effect indicators and others as accumulation indicators. Exposure to poor water quality may result in a reduction in parasite infection (Avenant-Oldewage 1994). Most studies involving accumulation indicators have focused on endoparasites, particularly acanthocephalans and cestodes. Sures et al. (1994) found that the acanthocephalan, *Pomphorhynchus laevis* infecting chub (*Leuciscus cephalus*), accumulated Pb in higher concentrations compared to the intestinal, muscle and liver tissues of the host. Similarly, *Acanthocephalus anguillae* accumulated Ag, Cd, Cu, Mn, and Pb in higher concentrations than their perch host (*Perca fluviatilis*) (Filipović et al. 2014). Retief et al. (2006) and Retief et al. (2009) in *Schyzocotyle acheilognathi* and Malek et al. (2007) in *Acanthobothrium* sp. and *Paraorigmatobothrium* sp. showed that concentrations of Cd and Pb were also higher in cestodes than in their hosts. Studies comparing trace-metal accumulation in ectoparasites are comparatively fewer. In two studies in two different monogenean species, Fe and Zn were accumulated in higher concentrations than in hosts’ muscle tissue (Gilbert et al. 2022; Nachev et al. 2022).

Regarding the regulation and detoxification of trace elements in parasites, compartmentalisation and sequestration occurred in hardened, sclerotised structures and eggshells in some taxa (Riggs et al. 1987; Sures et al. 1994; Shinn et al. 1995; Degger et al. 2009; Khalil et al. 2009; Gilbert and Avenant-Oldewage 2017, 2018). In endoparasites, metals became sequestered mostly on the eggshells. For instance, Riggs et al. (1987) in *S. acheilognathi* and Sures et al. (1997) in *Bothriocephalus scorpii* showed higher levels of Se, Cd, and Pb, respectively, in the gravid proglottids. Degger et al. (2009) and Khalil et al. (2009) confirmed that the metals bind on the eggshells of *S. acheilognathi* using fluorescence microscopy and X-ray microanalysis, respectively. As for the crustaceans, *Argulus japonicus* and *Argulus foliaceus*, metals are not sequestered on the eggshells, but instead in the sclerotised structures of the carapace (Haug et al. 2011; Gilbert and Avenant-Oldewage 2018). Furthermore, for another crustacean *Lamproglena clariae*, it was observed that exposure to metals resulted in a change to their metalloprotein expression (Ndaba et al. 2022) and a reduction in infection intensity in natural environments (Tsotetsi et al. 2004; Crafford and Avenant-Oldewage 2009; Pretorius and Avenant-Oldewage 2021). All previous studies have focused on attached adult female *L. clariae* (Ndaba et al. 2022; Pretorius and Avenant-Oldewage 2022). This can be attributed to the fact that only gravid female *L. clariae* parasitise the host fish, while all other life stages and adult males are free-living (Madanire-Moyo and Avenant-Oldewage 2013).

The current study reports on the biomineralisation and sequestration of elements in female *L. clariae* including the eggs and egg membrane. Specimens were collected from two different freshwater habitats in South Africa, which are variably impacted by pollution. The aims of the study were therefore to determine if metals accumulated by *L. clariae* become sequestered. Furthermore, if metals are sequestered, are they distributed variably through the body, and reflect levels in the environment.

## Materials and methods

**Fig.1:**
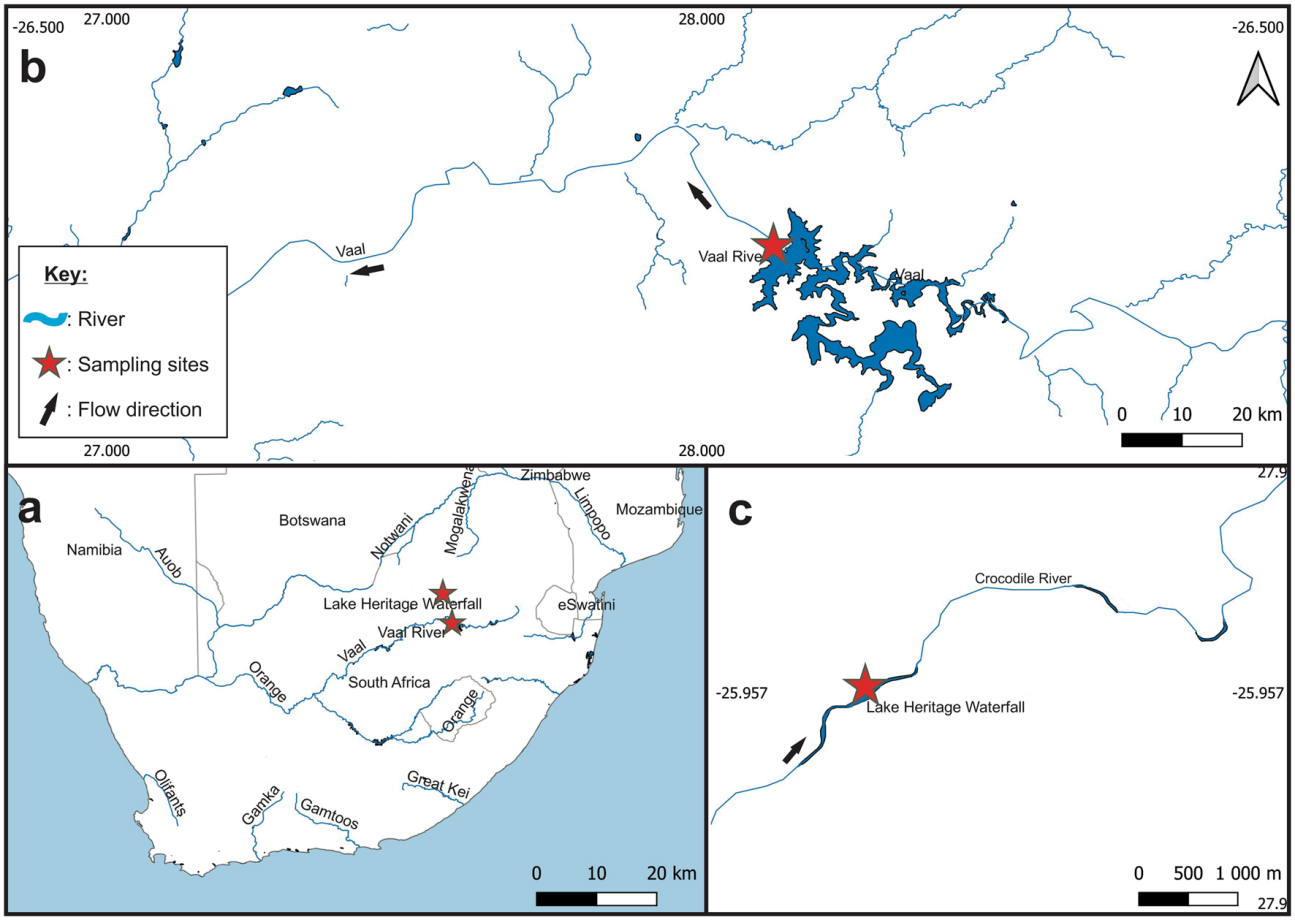
Sampling site locations in South Africa (**a**) for the Vaal River (**b**) and Crocodile River (**c**). Red stars () indicate sampling sites and black arrows (→) showing the direction of water flow in the rivers

Samples of adult female *L. clariae* were collected from the gills of *Clarias gariepinus* from Lake Heritage, along the Crocodile River (S25°57′16.923ʺ E 27°51′47.486ʺ) and in the Vaal River (S26°52′12.53ʺ E 28°7′14.09ʺ) below the Vaal Dam (Fig. 1). The Vaal River is known to be polluted (Crafford et al. 2011; Pretorius and Avenant-Oldewage 2021; Ndaba et al. 2022) and Lake Heritage is affected by acid mine drainage (Windisch et al. 2022). Gill nets were used to collect 10 *C. gariepinus* (stretched mesh size: 60–170 mm) per sampling site and transported to the field laboratory where they were weighed, measured, and euthanised by severing the spinal cord. The gills were removed and placed into a Petri dish with water from the site. The gills were inspected for *L. clariae* using a Zeiss Stemi 305 dissection microscope (Jena, Germany). Handling of the fish was done in accordance with guidelines by the South African National Animal Ethics Council and approved by the University of Johannesburg Ethics committee (Reference Number: 2021-04-01/Latief_Oldewage). All collections of the fish were done in accordance with the conditions of the permit issued by the Gauteng Department of Agriculture and Rural Development (permit number: CPE2-000140). Five *Lamproglena clariae* were collected, placed into 2 mL microcentrifuge tubes, flash frozen in liquid nitrogen, and later transferred for storage in a − 80 °C freezer (Evosafe-series VF720-86, Snijders Labs, The Netherlands) until sectioning.

### Cryosectioning

Specimens (*n = *5 per site) were embedded in optimal cutting temperature compound (Scigen Scientific, California, USA) and sectioned at 6 μm using a Reichert–Jung CryoCut E cryomicrotome operated at − 20 °C. Sections were mounted on cleaned microscope slides. Slides were cleaned with acid alcohol to remove trace elements. Sections on slides were air dried at room temperature for 30 min and stored at − 80 °C.

### Fluorescence microscopy

Sections were photobleached with a UV bulb (Philips TUV 30W, Holland), mounted in Phen-Green™ FL cell–permeant diacetate (Molecular Probes, Eugene, Oregon, United States of America) and sealed with clear nail vanish (Revlon) in the dark to prevent photodegradation. Sections were studied using a Zeiss Axioplan 2 epifluorescence microscope operated with Axiovision 4.3 software with rhodamine and DAPI band-pass filters (BP 365/12; excitation 490 nm; emission 520 nm).

### SEM–EDS analysis

Additional sections used for analysis of elements and to correlate with fluorescence results were dried in a desiccator cabinet (Kita-Ku, Osaka, Japan) and thereafter coated with carbon. The sections were analysed by point analysis at 20 keV using a Tescan Vega 3 scanning electron microscope (SEM) (Brno, Czech Republic) equipped with a X-Max50 energy-dispersive X-ray spectrometer (EDS) (Oxford Instruments, Halifax, England), operated by Aztec 2.1 software (Oxford Instruments, Halifax England) for Windows. Body tagmata and eggs were scanned for all elements. Only Mg, Al, Ca, Fe, Cu, and Zn were detected and levels were expressed in weight percentage (wt %).

### Statistical analysis

For the statistical analysis, IBM SPSS Version 28 Statistical package for Windows was used. To assess normality of the data for elements in wt %, the Levene’s test and histograms were used. As the data were not normally distributed, comparisons of element levels among tagmata were done using the Kruskal–Wallis test. In cases where there was a significant difference between tagmata a Mann–Whitney *U* test was used for pairwise comparisons. To test for differences in element levels between the exoskeleton and tissue in adults, and between egg contents and egg membrane, a Mann–Whitney *U* test was used. Differences in element levels in parasites from each site were tested using a Mann–Whitney *U* test. Bar charts were constructed for SEM–EDS data by log transforming the data (Ln [concentration + 1]), with the error bars displaying 95% confidence intervals. All statistical tests were assessed at a confidence level of 95%.

## Results

### Fluorescence microscopy

**Fig.2:**
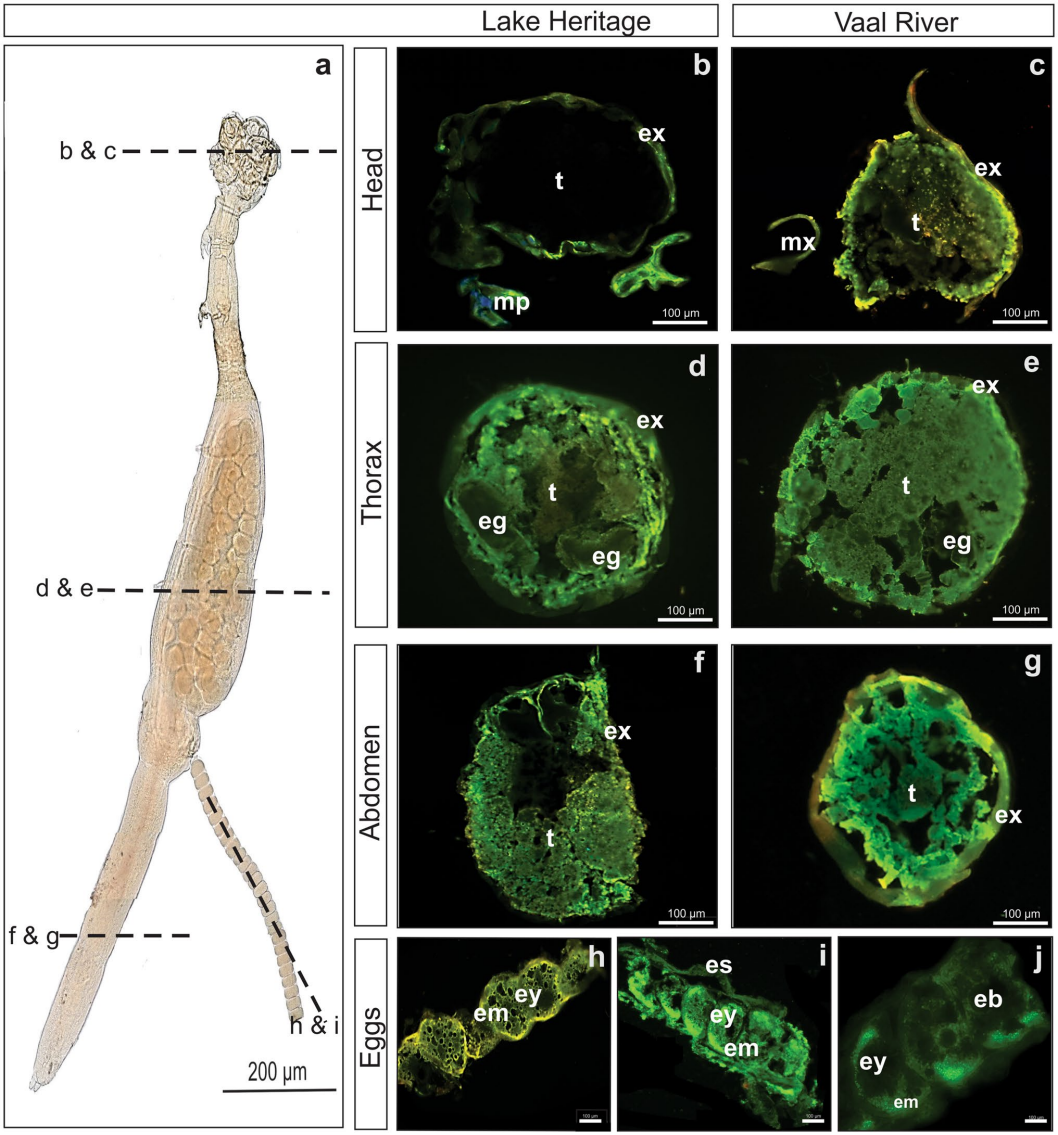
Light (**a**) and fluorescence (**b**–**i**) micrographs of *Lamproglena clariae* showing a whole mount (**a**) and sections through adults (**b**–**g**) and eggs (**h**–**i**). Dotted lines in **a** indicate section planes for micrographs **b**–**i**. Transverse sections through the head (**b** and **c**), thorax (**d** and **e**), and abdomen (**f** and **g**) show exoskeleton (ex), soft tissue (t), maxilliped (mp), maxilla (mx), and developing eggs (eg). Longitudinal sections through the eggs (**h**–**j**) show the egg yolk (ey), egg membrane (em), egg sac (sc), and embryo (eb). Fluorescence of the Phen-green indicate a positive reaction for divalent cations and trace elements. Micrographs were taken utilizing the Zeiss band-pass filter set 01 (BP 365/12) at 490–520 nm

Sections of adult female *L. clariae* collected from Lake Heritage and the Vaal River showed brighter fluorescence in the exoskeleton compared to the tissue of all tagmata (Fig. 2b–g). Sections through the egg sacs indicated brighter fluorescence of the egg yolk than the egg membrane (Fig. 2h–i), within eggs brighter signals were observed for yolk droplets (Fig. 2j). Furthermore, eggs within the thorax showed a weaker fluorescence signal in the membrane and yolk than those in the egg sacs (Fig. 2d, e, h and i). The egg sacs from Lake Heritage showed intense yellow fluorescence, whereas eggs from the Vaal River showed an intense green signal (Fig. 2h and i). Although specimens were collected from different sites, no differences were observed when comparing fluorescence signals of body sections.

### EDS analysis

**Fig. 3:**
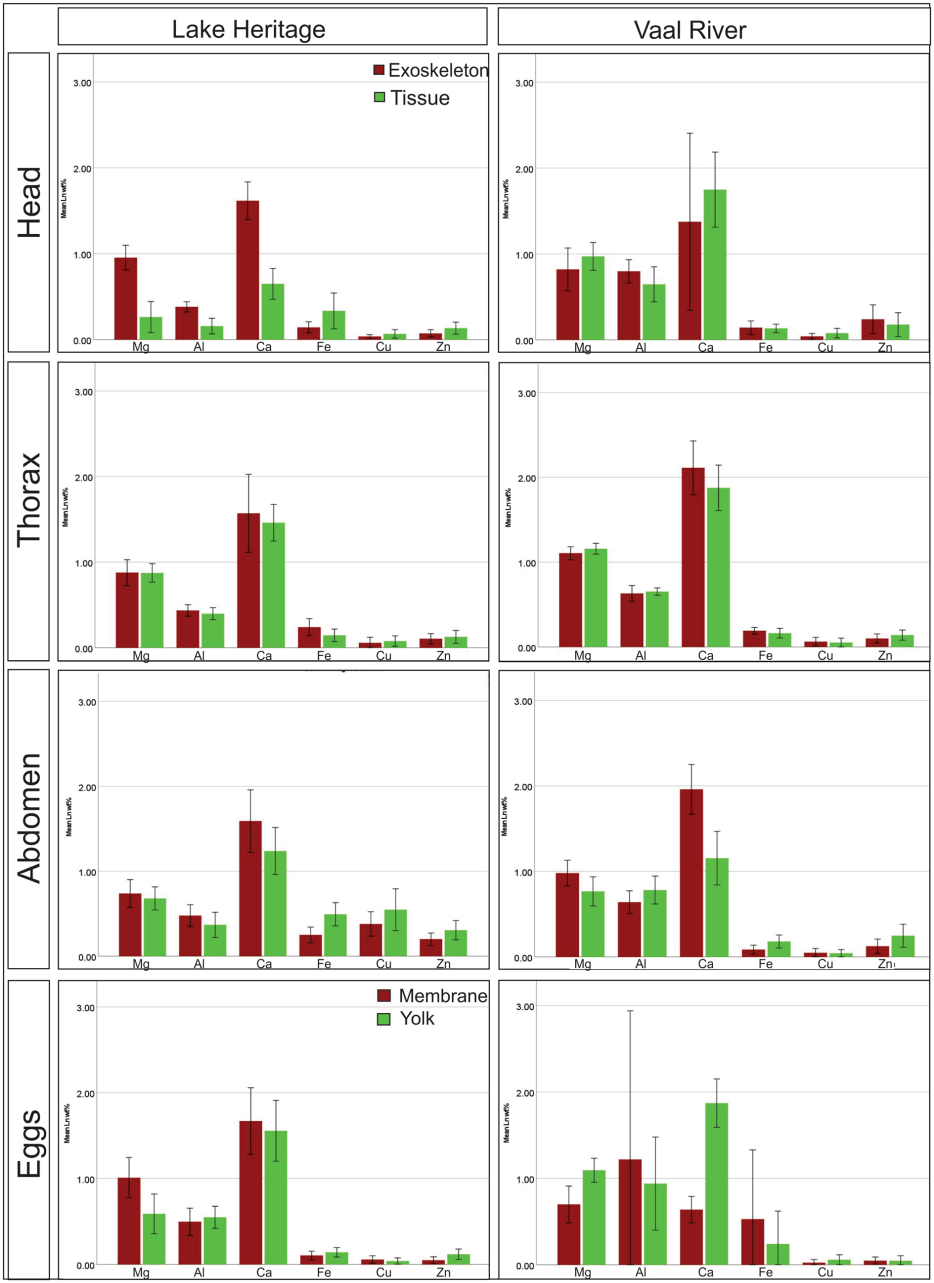
Bar graphs showing the mean for the log transformed weight percentages (wt%) for elements detected in sections of the head, thorax, abdomen, and eggs of *Lamproglena clariae* by SEM/EDS analysis for samples collected at Lake Heritage and the Vaal River. Red bars indicate elements found in the exoskeleton, while green bars indicate elements found in the tissue. The error bars indicate 95% confidence intervals

The presence of Mg, Al, Ca, Fe, Cu, and Zn was confirmed in all sections of tagmata and eggs (Fig. 3). The distribution of elements among the different tagmata, and the exoskeleton and tissue of the head, thorax, and abdomen varied. It also varied between the sites. Element concentrations in samples from Lake Heritage differed significantly in tissue between tagmata (Kruskal–Wallis for all elements; *p < *0.05). While, in the exoskeleton, only levels of Fe, Cu, and Zn were significantly different among tagmata (Kruskal–Wallis Fe, Cu and Zn; *p < *0.05). Comparing the exoskeleton to the tissue of all tagmata Al (Mann–Whitney *U* test: *Z = *− 2.12, *p = *0.034), Mg (Mann–Whitney *U* test: *Z = *− 4.29, *p < *0.001) and Ca (Mann–Whitney *U* test: *Z = *− 4.48, *p < *0.001) were higher. Whereas Cu (Mann–Whitney *U* test: *Z = *− 0.11, *p = *0.916) and Zn (Mann–Whitney *U* test: *Z = *− 1.78, *p = *0.075) were higher in the tissue than the exoskeleton for all tagmata, and Fe (Mann–Whitney *U* test: *Z = *− 1.94, *p = *0.052) was only higher in the exoskeleton of the thorax.

For specimens from the Vaal River, distribution of metals among the tagmata varied; for the tissue Al, Mg, Ca, and Zn (Kruskal–Wallis; *p < *0.05), while for the exoskeleton, Al, Fe, Mg, and Ca (Kruskall-Wallis; *p < *0.05) were significantly different. Higher levels of Al (Mann–Whitney *U* test: *Z = *− 0.31, *p = *0.755) and Fe (Mann–Whitney *U* test: *Z = *− 1.13, *p = *0.258) were detected in the exoskeleton of the head and thorax, but in the abdomen levels for both elements were higher in the tissue. The only element which showed similar accumulation patterns was Ca (Mann–Whitney *U* test: *Z = *− 1.10, *p = *0.272) which was consistently higher in the exoskeleton. Levels of Mg (Mann–Whitney *U* test: *Z = *− 1.78, *p = *0.075) showed opposite trends to Al and Fe, in that higher levels were detected in the tissue of the head and thorax, and in the abdomen levels were also higher in the exoskeleton than the tissue. For Zn (Mann–Whitney *U* test: *Z = *− 0.79, *p = *0.431), higher levels were detected in the exoskeleton of the head than in the tissue, but in the thorax and abdomen levels were higher in the tissue than the exoskeleton. Levels of Cu (Mann–Whitney *U* test: *Z = *− 0.62, *p = *0.537) showed no differences between exoskeleton and tissue in all body tagmata.

Element distribution within the eggs from both sampling sites showed no difference in levels of Cu (Mann–Whitney *U* test: *Z = *− 0.04, *p = *0.970) between the egg membrane and the egg contents. However, Al (Mann–Whitney *U* test: *Z = *− 0.09, *p = *0.929), Fe (Mann–Whitney *U* test: *Z = *− 0.12, *p = *0.902), and Zn (Mann–Whitney *U* test: *Z = *− 1.28, *p = *0.199) were higher in the yolk than the egg membrane for *L. clariae* from Lake Heritage, while at the Vaal River, these metals were higher in the egg membrane than the yolk. Mg (Mann–Whitney *U* test: *Z = *− 0.01, *p = *0.990) and Ca (Mann–Whitney *U* test: *Z = *− 0.90, *p = *0.366) were higher in the yolk in parasites collected at the Vaal River, whereas at Lake Heritage, these metals were higher in the egg membrane.

## Discussion

In *L. clariae* treatment with Phen-Green, resulted in more intense fluorescence of the exoskeleton compared to internal tissues. The intensity of the fluorescent signal of Phen-Green is related to the concentration and type of ion present (Johnson and Michelle 2010), and therefore, areas of the body which are more mineralised will show brighter fluorescence. Similarly, in *A. japonicus,* Gilbert and Avenant-Oldewage (2018) reported a more intense fluorescence signal in the sclerotised parts of the exoskeleton. Higher metal levels in the exoskeleton of *L. clariae* were confirmed using SEM–EDS and Mg, Al, Ca, and Fe were higher in the exoskeleton compared to the internal tissues. It is well known that the sclerotised parts of the exoskeleton in invertebrates incorporates elements (Quicke et al. 2004) and offers protection to the internal structures of crustaceans (Olesen 2013). Metals, such as Zn, occur in the cutting edges of the mandibles and maxillae of *Atta sexdens* (leaf-cutting ants) and the jaws of *Nereis limbata* (marine polychaete) (Schofield et al. 2002; Lichtenegger et al. 2003).

In addition to the protective and functional aspects associated with biomineralisation of the exoskeleton, it functions in reducing trace element and metal burdens (Keteles and Fleeger 2001; Bergey and Weis 2007). Keteles and Fleeger (2001) reported that Cd concentrations were reduced in the grass shrimp, *Palaeminetes pugio*, following ecdysis. Similarly, Bergey and Weis (2007) showed that crab, *Uca pugnax* from contaminated sites had higher Pb concentrations in the exoskeleton compared to the internal tissues and therefore concluded that moulting can significantly reduce body burdens. Despite the current results showing that the exoskeleton of *L. clariae* is more enriched with some elements than the internal tissues, it is unlikely that the exoskeleton in adult females plays a significant role in metal regulation. This is because it is unlikely that moulting occurs in permanently attached adult females of *L. clariae*. Therefore, elements are likely deposited as a means of providing support to the exoskeleton, but this may be a limited means of regulating metals.

Metals are deposited on the eggs as a means of reducing body burdens in some parasites (Sures et al. 1997; Degger et al. 2009; Khalil et al. 2009; Gilbert and Avenant-Oldewage 2017, 2018). However, in adult female *L. clariae* fluorescence and SEM–EDS results confirmed that elements were deposited inside the egg, specifically in the yolk. Sequestration in the eggs is supported by the fact that elements, such as Al, Mg, Ca, Fe, and Zn, were higher in the eggs compared to sections of adult parasites from both sites. In cestodes, Sures et al. (1997) showed that gravid proglottids had higher Pb levels, and this was later confirmed to be bound onto the eggshells (Degger et al. 2009; Khalil et al. 2009).

However, in ectoparasites, Gilbert & Avenant-Oldewage (2017, 2018) showed that the eggshells in *P. ichthyoxanthon* and *A. japonicus* lack elements, rather metals sequestered to the vitellaria in *P. ichthyoxanthon* (Gilbert and Avenant-Oldewage 2017) and gelatinous layer around the eggshell in *A. japonicus* (Gilbert and Avenant-Oldewage 2018). In the present study, a positive signal for yolk droplets within the eggs of *L. clariae* suggest that similar to *P. ichthyoxanthon,* elements become associated with yolk.

Comparisons between parasites from Lake Heritage and the Vaal River showed variable element levels in sections. Differences corroborated published results on dissolved element levels from each site for some elements. For instance, Zn and Fe levels in the eggs of *L. clariae* from Lake Heritage were higher than in specimens from the Vaal River, which matched the differences in levels of these elements in the water and was conducted parallel to this study (Ndaba et al. 2022; Windisch et al. 2022). Such similarities can be related to the fact that *L. clariae* is an ectoparasite and therefore is exposed to and accumulates elements present in the water and/or host blood.

Therefore, in conclusion, results from the present study demonstrated that *L. clariae* accumulates elements present in the macroenvironment, and reflect levels dissolved in the water. Like other crustaceans, *L. clariae* deposits metals in the exoskeleton where they assist in providing support to the exoskeleton. In terms of regulating metals, it is unlikely that elements are released during moulting of the exoskeleton in adult female *L. clariae*. Rather, the current results suggest that elements become sequestered to yolk. Higher levels of elements in the yolk may become transferred to and accumulated by the nauplii. These elements could then bind to the exoskeleton of the nauplii and be released when they moult. Therefore, one way that adult *L. clariae* females could regulate element body burdens is through sequestration into the egg.

## Data Availability

All data generated or analyzed during this study are included in the published article. Raw data can be requested from the first author on reasonable request.
